# Not Every Size Fits All: Surgical Corridors for Clival and Cervical Chordomas—A Systematic Review of the Literature and Illustrative Cases

**DOI:** 10.3390/jcm13175052

**Published:** 2024-08-26

**Authors:** Rosario Maugeri, Lapo Bonosi, Lara Brunasso, Roberta Costanzo, Samuele Santi, Francesco Signorelli, Domenico Gerardo Iacopino, Massimiliano Visocchi

**Affiliations:** 1 Neurosurgical Clinic, AOUP “Paolo Giaccone”, Post Graduate Residency Program in Neurologic Surgery, Department of Experimental Biomedicine and Clinical Neurosciences, School of Medicine, University of Palermo, Via del Vespro 127, 90127 Palermo, Italy; rosario.maugeri1977@gmail.com (R.M.); lapo-bonosi@community.unipa.it (L.B.); roberta.costanzo@commuity.unipa.it (R.C.); gerardo.iacopino@gmail.com (D.G.I.); 2 Department of Neurosurgery, Fondazione Policlinico Agostino Gemelli IRCCS, Largo Agostino Gemelli 8, 00168 Rome, Italy; samuele.santi01@unicatt.it (S.S.); francesco.signorelli@policlinicogemelli.it (F.S.); massimiliano.visocchi@policlinicogemelli.it (M.V.); 3 Institute of Neurosurgery, Catholic University of the Sacred Heart, Largo Francesco Vito 1, 00168 Rome, Italy

**Keywords:** chordomas, clival, cervical, CVJ, transoral approach, endonasal approach, retropharyngeal approach

## Abstract

**Introduction.** Clival chordomas represent a rare but clinically significant subset of skull base tumors, characterized by a locally aggressive nature and a location in proximity to vital neurovascular structures. Surgical resection, often combined with adjuvant therapies, remains the cornerstone of clival chordoma treatment, and various approaches and techniques have evolved to maximize tumor removal while preserving neurological function. Recent advancements in skull base surgery, imaging, and adjuvant therapies have improved outcomes by reducing morbidity and thus enhancing long-term survival. **Methods and Results.** We have conducted a systematic review on PubMed/Medline following PRISMA guidelines regarding indications, the extent of resection (EOR), and complication rates. Then, we present three illustrative cases from our personal experience, which started 25 years ago with CVJ instrumentation procedures and 15 years ago with anterior decompressive transmucosal procedures performed with the aid of an operative microscope, an endoscope, and neuroradiological monitoring. **Conclusions.** Traditionally, the transoral approach (TOA) is the most frequently used corridor for accessing the lower clivus and the anterior craniovertebral junction (CVJ), without the need to mobilize or retract neural structures; however, it is associated with a high rate of complications. The endonasal approach (EEA) provides access to the anterior CVJ as well as to the lower, middle, and superior clivus, decreasing airway and swallowing morbidity, preserving palatal function, decreasing postoperative pain, and reducing the incidence of tracheostomy. The submandibular retropharyngeal approach (SRA) allows unique access to certain cervical chordomas, which is better suited when the lesion is located below the clivus and in the midline.

## 1. Introduction

Chordomas are rare, slow-growing tumors arising from remnants of the notochord. Due to their embryological origin, chordomas can be found along the vertebral axis, but they are most frequently encountered cranially at the clivus and craniovertebral junction (CVJ) and caudally at the sacrococcygeal junction [1]. At the CVJ, chordomas usually involve the clivus with a caudal extension to C1 and even to C2 in some cases. A report from the RARECARE Project estimates that the incidence of chordoma is 1 in 1,000,000 individuals and the prevalence is less than 1 in 100,000 [2]. According to the “Chordoma Foundation”, approximately 300 new cases of chordoma are diagnosed each year in the United States and about 700 throughout Europe. Chordomas of the skull base occur more frequently in younger patients, while spinal chordomas are more common later in life [3]. Although chordomas are considered to be slow-growing and histologically low-grade tumors, they show aggressive and destructive tumor behavior, and their high recurrence rate makes their clinical course similar to that of malignant tumors [1]. This is particularly true for clival chordomas, whose deep anatomic location and proximity to vital anatomic structures make surgical resection hard and challenging.

Radical surgery is widely considered the aim of the standard treatment as preferential primary en-bloc resection. Specifically, the extent of resection for chordomas is a recognized prognostic factor showing a positive impact on survival outcomes when associated with high-dose adjuvant radiotherapy (RT). Nevertheless, the recent literature still reports only a modest benefit on long-term survival, and despite maximal or gross total tumor resection (GTR) followed by RT, the recurrence rate remains high with estimated progression-free survival (PFS) rates of 65% and 32% at 5 and 10 years, respectively [4]. Besides that, especially for clival chordomas, postoperative significant morbidity and long-term sequelae can result in influencing the final patients’ outcome.

Over the years, evolving surgical techniques have played a pivotal role in enhancing the precision and safety of CVJ lesion resections [5,6]. Among these, the endoscopic endonasal (EEA), transoral (TOA), and retropharyngeal approaches have emerged as promising and less invasive techniques, offering unique advantages in accessing and resecting these challenging tumors.

Herein, we provide a comprehensive review and comparative analysis of the EEA, TOA, and retropharyngeal surgical approaches for clival and upper cervical chordomas, starting from the analysis of the historical context of chordoma management and delving into the evolution of these innovative techniques, highlighting the strengths and limitations of each. Furthermore, we discuss three illustrative cases from the personal experience of the senior author (M.V.), alongside the impact of technological advancements, such as high-definition endoscopy and neuronavigation, on improving surgical outcomes. As we enter an era of personalized medicine, optimizing chordoma treatment requires a nuanced understanding of surgical techniques and their outcomes.

This article contributes to the existing body of knowledge by synthesizing current evidence and providing insights into the evolving landscape of surgical management for clival and cervical chordomas.

## 2. Materials and Methods

Preferred Reporting Items for Systematic Reviews and Meta-Analyses guidelines (PRISMA) were followed to conduct this systematic literature review. A systematic literature search on PubMed/Medline for all studies investigating indications, the extent of resection (EOR), and the complications rates of TOA, EEA, and retropharyngeal approaches for clival chordomas was performed up to the 14th of October 2023 without backward limits. The following MeSH terms “transoral approach” AND “chordoma” AND “transnasal approach” AND “skull base chordoma” AND “endonasal approach” AND “retropharyngeal approach” were used. To avoid the potential omission of relevant studies, we also manually screened reference lists of articles included in previous systematic reviews and meta-analyses regarding this topic. Duplicate articles were eliminated using Microsoft Excel 16.37. The research strategy initially relied on title and abstract analysis. The article’s full text was retrieved for further investigation if the title and abstract met the inclusion criteria. The data collection process was conducted without using any automated tools. According to the following criteria, all articles were identified by three reviewers (L.Br., R.C, and L.Bo.) separately. In case of a discrepancy, the paper was discussed until a consensus among the investigator authors was reached. No ethical approval was required for this study.

The articles were selected according to the following inclusion criteria:

- Full article in English;
- Studies analyzing more than 5 patients;
- Case series, retrospective studies, and prospective studies;
- Patients affected by skull base and/or cervical chordomas treated with a transoral, endonasal, or retropharyngeal approach;
- Studies evaluating EOR and complication rate;

The exclusion criteria were the following:

- Articles not in English;
- Editorials, books, case reports, systematic reviews, and meta-analyses;
- Anatomical studies:
- Studies focusing on other surgical approaches.

## 3. Results

**Figure 1:**
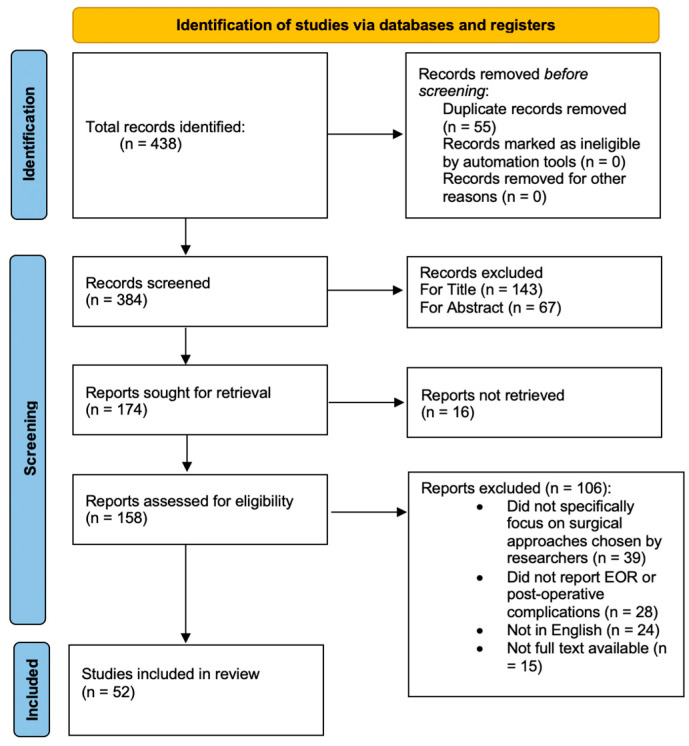
Preferred Reporting Items for Systematic Reviews and Meta-Analyses (PRISMA) flow diagram for the present systematic review of the literature about indications, the extent of resection, and complication rates of transoral, endoscopic endonasal, and retropharyngeal approaches for clival chordomas.

Our initial research identified a total of 438 articles. We excluded 55 duplicate articles. After further screening based upon title and abstract reading, 210 articles were removed. Finally, after a full text reading, 110 articles were excluded because they focused on other surgical approaches different from those chosen by the researchers (39 articles); they did not report information about outcomes, EOR, or complications (28 articles); or because they were not written in the English language (24). Finally, for 15 papers, we were unable to locate the full text. So, we included 52 studies in our systematic review, according to the PRISMA flow diagram inclusion criteria (Figure 1). Demographic data of the included studies are presented in Table 1. Technical aspects, outcomes, and complications related to the surgical approach are extrapolated in Table 2.

### 3.1. Study Characteristics and Data Analysis

Of the 52 studies included in the review, 19 (48.7%) were conducted in Europe, 15 (28.8%) in the US and North America, 15 (28.8%) in Asia, 2 (3.8%) in South America, and only 1 (1.9%) in Australia. The selected articles analyzed a total of 3515 patients, including 1673 specifically with chordomas of the basilar and upper cervical spine. The total number of males was 639, with an M/F ratio of 0.61. In 13 studies, the number of males or the M/F ratio was not reported. The mean age of the evaluated population was 47.24 years (range 4–84). In two studies, biographical data were not available, and in eight, they were not segregable from patients evaluated but not with chordomas. Furthermore, 39 papers focused on the EEA and its various extensions/variations, 3 on the TOA, 2 on the retropharyngeal approach, and 8 on the comparison of various surgical approaches. The average follow-up time was 32.16 months.

Reported symptoms included visual field and oculomotion disorders (n = 637, where the most frequently involved was VI cranial nerve), headaches (n = 302), cervicalgia (n = 107) signs of myelopathy and postural deficits (n = 71), other cranial nerve disorders (in 220 cases), and finally other onset symptoms in 98 cases (hormonal disorders, infections, CSF leaks, and airway or nasal obstructions).

Localization was reported at the clivus in 38 studies, with involvement in its upper portion in 384 cases, middle in 225, lower in 296, and the upper cervical spine in 258 cases. In 14 studies, the location of chordomas was not explicitly reported or evaluated; 26 studies evaluated the intramural extension present in 370 cases.

All included studies evaluated the EOR. In 823 cases, a GTR was achieved (>90%), in 458 (70–90%) and in 125 cases, a partial resection (<70%). In 13 studies, no CSF leak occurred; 8 studies did not explicitly report this complication; in 2 studies, these data were not available; and 29 papers evaluated its incidence, reporting 145 cases of CSF leak. Among other postoperative complications, the most frequent was paralysis (n = 107), transient or permanent, of one or more cranial nerves, followed by ischemic or hemorrhagic events (n = 30), hydrocephalus (n = 11), infections (n = 47), and hormonal disorders (n = 6). In 17 studies, primary surgery was performed, while 21 studies examined both cases of primary surgery and operations upon relapses (“second-time surgery”).

Finally, 11 studies evaluated the days of hospitalization after surgery, with an average of 10.2 days (range 2–36 days).

### 3.2. First Case: Two-Staged Submandibular Retropharyngeal and Endoscopic Endonasal Approach

**Figure 2:**
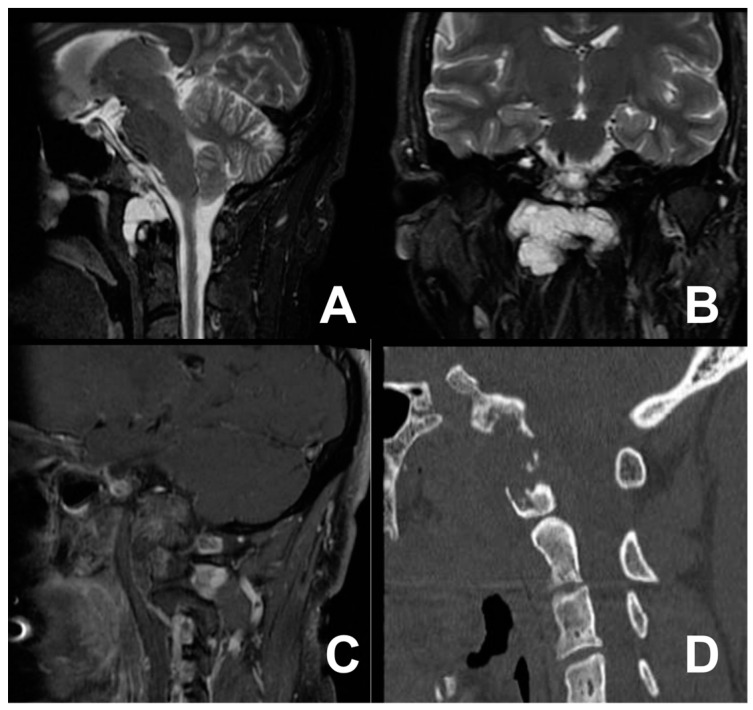
T2-weighted MRI scan in the sagittal (**A**) and coronal views (**B**) and T1 sagittal post-contrast image (**C**) showing the cervical tumor anteriorly extending toward the occipital condyles, involving the right anterior arch of the atlas, with spongy bone replacement and consequential destruction of the cortical and right side of the clivus (**D**).

**Figure 3:**
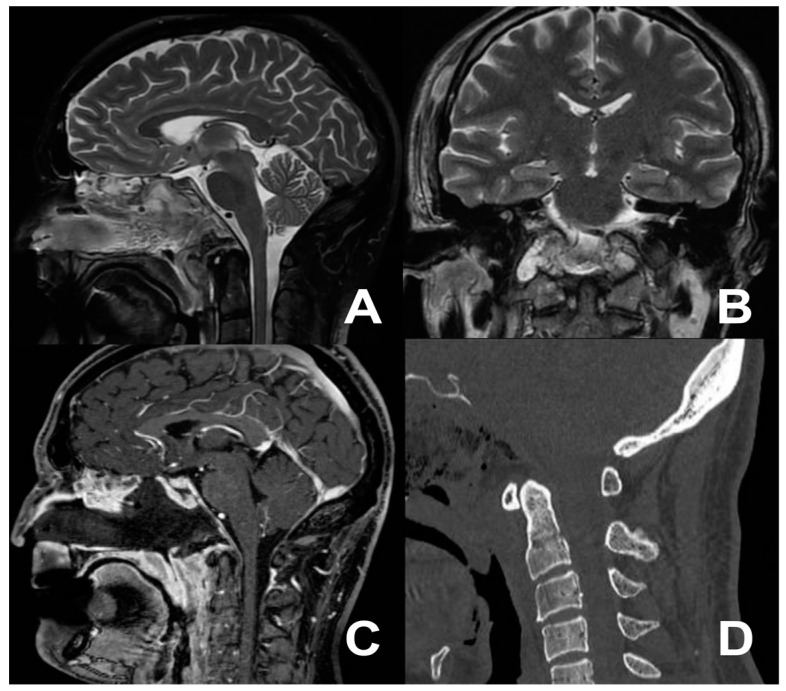
Postoperative MRI (**A**–**C**) and CT scan (**D**) showing the extent of tumor removal.

A 44-year-old male with a documented history of Bechet’s disease presented with severe migraines and recurrent dizziness. Diagnostic investigations included a cervical contrast-enhanced computed tomography (CT) scan and cervical contrast-enhanced magnetic resonance imaging (MRI), revealing the presence of a mass highly compatible with a diagnosis of chordoma. The tumor exhibited immediate extension anterior to the occipital condyles, involving the right anterior arch of the atlas with spongy bone replacement and the consequential destruction of the cortical and right side of the clivus (Figure 2). The patient complained of neck stiffness and pain. Considering the patient’s autoimmune pathology, at first, we decided to avoid a transmucosal approach. Therefore, a right submandibular approach was performed. Regrettably, total control of the lesion proved elusive, leading to the strategic decision to perform an extended biopsy. A histopathology examination confirmed the chordoma diagnosis. Postoperatively, the patient developed transient dysphagia for solids and liquids, which spontaneously resolved within a week. Three weeks after the first procedure, an EEA was performed, successfully facilitating the total removal of the previously identified lesion, confirmed by postoperative imaging (Figure 3). The patient was discharged on the sixth postoperative day, with no new neurological deficits.

### 3.3. Second Case: One-Stage Combined Submandibular Retropharyngeal Approach and Posterior Midline C1-C2 Fusion

**Figure 4:**
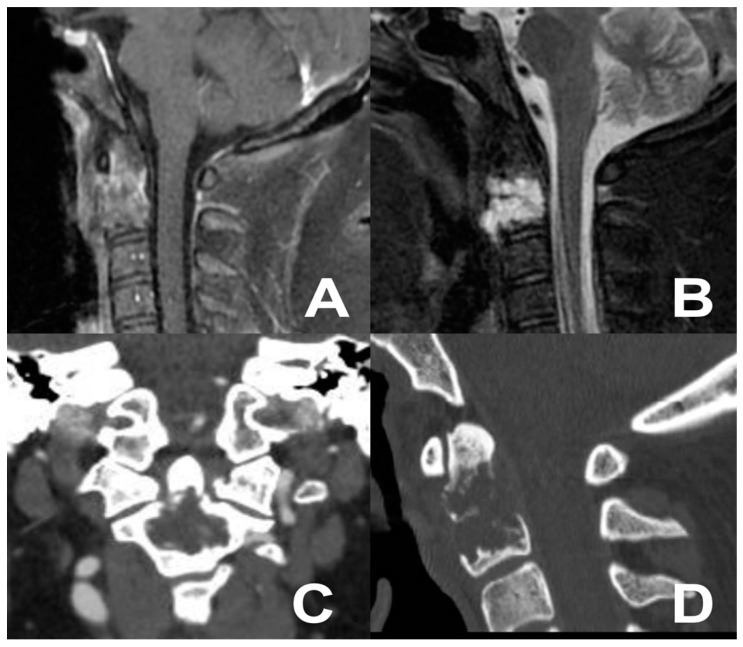
Cervical MRI (**A**,**B**) and CT scan (**C**,**D**) showing the osteolytic lesion of the C2 vertebral body.

**Figure 5:**
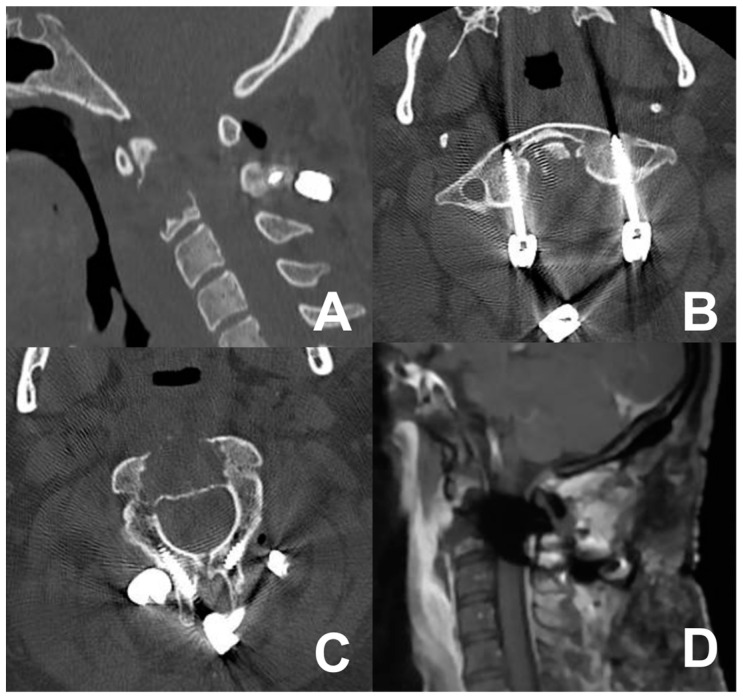
Postoperative CT scan (**A**–**C**) and MRI (**D**) showing the extent of bone removal, tumor resection, and posterior fixation.

A 39-year-old male presented with neck pain following trauma. An initial cervical X-ray revealed an osteolytic lesion of the C2 vertebral body. Subsequent cervical contrast-enhanced MRI identified an osteolytic lesion of the C2 vertebral body (Figure 4). An adjunctive PET-CT scan revealed a metabolically active glucose lesion in the cervical region. The patient underwent a one-stage combined submandibular retropharyngeal approach for lesion excision and a posterior midline approach for C1-C2 arthrodesis with rods and screws. The postoperative course was uneventful, without additional neurological deficits. Follow-up cervical MRI and CT revealed the successful lesion removal and appropriate positioning of synthesis materials (Figure 5). A chordoma was diagnosed by definitive histopathological examination.

### 3.4. Third Case: Two-Staged Transoral Approach and Posterior Midline Occipitocervical Fusion—Subtemporal/Infratemporal and Retrosigmoid Approaches for Tumor Relapse

**Figure 6:**
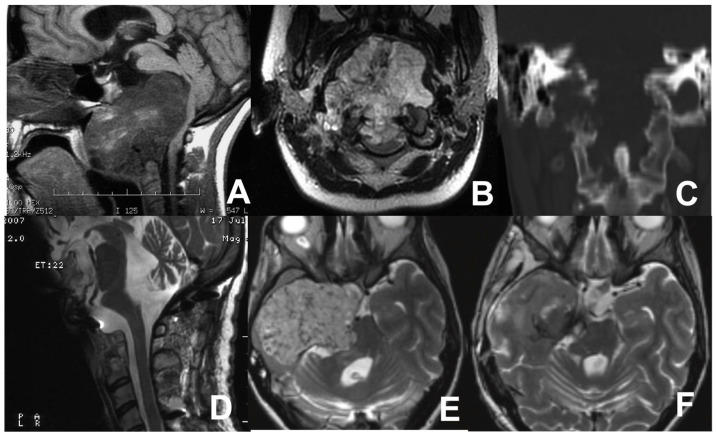
MRI sagittal (**A**) and coronal (**B**) views showing the huge chordoma in the lower third of the clivus, extending to the body of C2, causing posterior displacement of the brainstem, and compressing the nasopharynx. CT scan (**C**) showing osseous erosion of the occipital condyles, anterior arch of C1, and a portion of the C2 body. Postoperative MR scan after first operation (**D**). Pre- (**E**) and postoperative (**F**) axial MRI scans after the tumor relapse involving the clivus, sphenoid region, right petrous bone, and temporal fossa with severe compression of the brainstem.

A 27-year-old female with a history of progressive head and neck pain, difficulty swallowing, and dysarthria presented to our attention. A neurological examination revealed IX and XII cranial nerve deficits, hyper-reflexia, and ataxia. A contrast-enhanced brain and cervical MRI was performed, revealing a sizable mass in the lower third of the clivus extending to the body of C2, causing the posterior displacement of the brainstem and compressing the nasopharynx, initially suggestive of chordoma. A further CT scan demonstrated the osseous erosion of the occipital condyles, anterior arch of C1, and a portion of the C2 body (Figure 6). A TOA was initially performed. Then, a second-stage, posterior stabilization of C1-C2-C3 through rods and screws was accomplished. Postoperative MRI confirmed the partial removal of the lesion. The progressive re-growth of the residual chordoma was documented on follow-up MRI, prompting the decision to proceed with a second surgical intervention 3 years after the previous one. Upon admission, the patient presented cachexia, left hemiparesis, midline and right hemispheric cerebellar syndrome, and palsy of right cranial nerves from V to XII. Preoperative MRI revealed a significant increase in the size of the lesion involving the clivus, sphenoid region, right petrous bone, and temporal fossa with severe compression of the brainstem. Tracheostomy and gastrostomy were performed.

A two-stage surgical procedure was performed, employing subtemporal/infratemporal and retrosigmoid approaches. The patient underwent extensive tumor resection and decompression of the temporal lobe and brainstem. A remarkable improvement in the patient’s neurological conditions was documented. The closure of the gastrostomy occurred prior to discharge, and the tracheostomy was removed one month later.

## 4. Discussion

Largely accepted reports indicate that the maximal safe removal of clival chordomas followed by radiotherapy provides the best long-term survival [3]. It is evident that the grade of resection is predominantly dependent on the size and the extension of the tumor, and while small and well-circumscribed tumors within the clivus can easily be surgically resected, in patients with widely extended tumors, GTR can rarely be achieved. Due to the heterogeneous characteristics of included patients in retrospective studies, the evidence from the literature is still poor [57,58]. Most chordomas involve the clivus with a variety of tumor extensions from local infiltration to C1 and/or C2 and/or skull base structures, e.g., cavernous sinus. With the use of surgical microscopes in the 1960s, the TOA and sublabial techniques were developed [59]. Since the 1960s, the trans-sphenoidal approach has been the mainstay of chordoma surgery with the natural nasal corridor advocated as the most direct route to the clivus [60]. The implementation of endoscopic techniques has offered advantages and less invasive options, and several recent studies have reported their safety and effectiveness [61,62,63].

### 4.1. Transoral Approach

The midline transoral–transpharyngeal approach is widely explored in the literature as a convenient route for accessing extradural midline lesions of the CVJ and upper cervical spine [55,64]. The lower clivus, atlas, and part of the axis are readily accessible via this route, and TOA can be combined with palatal or mandibular splitting procedures for both additional rostral and caudal exposure. Some conditions like rheumatoid arthritis, mandibular disorders, or older age can limit the mouth’s opening, and a median glossotomy, circumglossal approach, or mandibular splitting procedure may be necessary to gain access. The standard TOA is performed through a transoral–transpalatal route, and velopharyngeal incompetence, hypernasal speech and nasal reflux, dental injury, edema or tongue necrosis, posterior pharyngeal wound dehiscence, and meningitis are the major potential complications [65]. Choi et al. [12] reported their vast experience with 97 patients. The most common operations performed were the standard TOA and “open-door” maxillotomies, and the latter was associated with greater complications such as nasal regurgitation. Wang et al. [66] reported their experience in treating three of eight patients with C1-C2 recurrent chordoma after RT with TOA combined with a posterior approach. In two patients, subtotal tumor removal was achieved and local recurrence was then documented during the follow-up period; in all patients, incision disunion was noted; and in two patients, CSF leaks were reported as postoperative complications. A large study about primary atlantoaxial bone tumors in the pediatric population by Menezes et al. [26] described five chordoma cases. TOA was the modality of choice for resecting a 14-year-old male’s and 8-year-old female’s C2 and C1 chordomas, respectively, and a 7-year-old male’s C1-2 to clivus chordoma. TOA was always followed by stabilization or fixation for consequent spinal instability. Two patients received LINAC irradiation, and two other patients elected to undergo proton beam treatment. No recurrence was seen, and no infections were detected in any patients. A CSF leak was reported for the C1 chordoma with intradural invasion, repaired with fascial and fat graft. The authors claimed that a gross resection should be followed with radiation therapy for chordomas also in the pediatric population.

Despite direct access for approaching the clival and CVJ area, the TOA has several limitations. The surgical field of a traditional TOA is limited, contributing to the incomplete resection of large chordomas. Although several modifications to improve the exposure of standard TOA, such as extended incision with a U-shaped flap, the transoral-mandibulotomy–glossotomy approach, or the transmaxillary–transmandibular approach, the germ-laden oral cavity and wide and invasive tissue dissection place a challenging problem for surgical wound healing, especially for those patients with previous RT [66]. The concomitant advent of CSF leaks can lead to potentially fatal complications. Moreover, some patients may also need tracheostomy before surgical procedures.

The innovative association between the traditional open exposure of the TOA and the advantages of improved visualization through the use of endoscopy have become popular topics as an endoscopic-assisted transoral approach also known as the endo-oral approach [62,63,64].

### 4.2. Anterior Retropharyngeal Approach

In cases of chordomas where a wide exposure to achieve total resection is required, the high anterior cervical approach is advocated for the adequate decompression of the cervico-medullary junction and, contextually, the possibility of anterior cervical fixation/fusion. The retropharyngeal approach is reported as a favorable route to treat tumor lesions of the C2 vertebral body through a horizontal incision; it can be considered as an alternative to the TOA for the same direct access to the anterior part of the upper cervical spine while preserving the mucosa of the oropharynx, lowering the rate of potential pharyngeal complications, avoiding entry into the bacteria-contaminated environment, and offering a greater degree of bilateral exposure than the transoral route [65]. In addition, no tracheostomy is required in this procedure. Neurosurgeons are familiar with the anterior cervical approach with traditional instrumentation used for the anterior retropharyngeal approach.

The complex anatomy involved in this access represents the main disadvantage of this approach; the risk of injury to the submandibular gland, the facial artery and vein, and the hypoglossal and superior laryngeal nerves must be taken into account [52,67]. Yang et al. [47] reported two cases of C2-C3 chordomas approached through a retropharyngeal approach with postoperative difficulties in swallowing in both cases and a CSF cyst in one case. Both chordomas relapsed at 13 and 18 months postoperatively, and one patient deteriorated with high paraplegia and died of respiratory failure.

The classic SRA is a demanding approach that requires detailed knowledge of submandibular region anatomy and ENT surgical support, and it carries a considerable risk of complications. Consequently, the SRA is rarely performed. A recent simplification of the approach is based on the identification of a natural anatomical corridor between the two key landmarks: the inferior belly of the submandibular gland superiorly and the greater horn of the hyoid bone infero-medially [68].

### 4.3. Endoscopic Endonasal Approach

Historically, endoscopic endonasal transsphenoidal techniques were primarily utilized for treating sellar lesions due to their midline access, which facilitated reaching para-/retro- and suprasellar spaces without the need for brain retraction or invasive transcranial approaches [69]. Over the past two decades, this approach has been extended to address other regions, such as the clivus and the CVJ, offering a broader operative view through a less invasive route, and it was particularly evident for treating clival chordomas. In such cases, the EEA employing a straight route and a wide angle is considered the most advocated solution [51,56]. The endoscopic endonasal transclival approach is a binostril approach usually performed to facilitate four-handed surgery, enhancing the ability to obtain a wider corridor and more easily identify major anatomical landmarks [70]. Following the nasal phase involving middle and superior turbinectomy, the preparation of a nasoseptal flap is undertaken, along with a wide sphenoidotomy and posterior nasal septectomy. Subsequent steps in the procedure are tailored according to tumor location and extension [27]. The reconstruction phase is essential to avoid postoperative infections and CSF leaks and might be performed using a nasoseptal vascularized flap, duroplasty, biologic glue, and, whenever necessary, fat grafts [34,36].

An extended EEA may provide several advantages for clival lesions including the absence of brain retraction with a wider angle and a comprehensive view of intra- and extradural spaces, potentially influencing the EOR [36]. Additionally, this approach allows access to areas that may be inaccessible through a transcranial route and is associated with a lower incidence of bacterial infection, dysphagia, or speech disorders compared to oropharyngeal or soft palate approaches [36,71]. Clival chordomas can be extradural or intradural and may be in the upper, middle, or lower clivus or extend along the entire clivus (holoclivus).

1. Extradural–Upper Clivus: These clival chordomas are typically in the sellar region and confined by the cavernous sinus. In cases of retrosellar invasion, the extradural transposition of the pituitary gland is employed. Additionally, the resection of the posterior clinoid is usually performed. Tumors in the upper clivus have a higher resection rate [16,69].
2. Extradural–Middle Clivus: The paraclival carotid serves as the boundary for this region, accessible through the petrous apex area [10,22].
3. Extradural–Lower Clivus: The boundaries are represented by the condyles. However, employing an EEA in this area is relatively contraindicated, especially in the case of lesions near the lower cranial nerves located posterior to the occipital condyle [22].
4. Intradural: These lesions lack defined boundaries and require management through different corridors (e.g., infra-chiasmatic in the upper clivus) being careful around damage to the VI nerve [10].

Shidoh et al. [71]—on the surgical frame—and Visocchi et al.—on cadaveric dissections [64]—compared the EEA to the TOA. The latter was primarily used to access lesions around the CVJ, providing better exposure of the clival region in sagittal and coronal planes, as proven even in cadaveric studies. However, the lateral extension of lesions might hinder the resection rate due to limitations posed by the pterygoid process and the atlanto-occipital joint. Therefore, in many procedures, the EEA has replaced the TOA due to reduced dead angles with the use of an endoscope, allowing GTR in most upper and middle clival lesions but posing challenges in reaching lower clival lesions due to the presence of the hard palate [71]. A combined approach (EEA + TOA) could potentially ensure a more radical and less invasive surgery [71].

The EEA has revolutionized the management of clival chordomas, offering a less invasive yet highly effective surgical method with reduced postoperative complications and shorter hospital stays. This approach has gradually been introduced into the pediatric population, facilitated by innovative instrumentation and the use of neuronavigation, thereby minimizing brain retraction and manipulation and leading to decreased hospital stays and postoperative complications, ultimately improving their quality of life [10]. Nevertheless, EEA has many drawbacks related to the smaller surgical domain of the lower CVJ and the risk of velo-palatal insufficiency [62].

### 4.4. Limitations and Future Directions

This systematic review has some limitations that warrant consideration. First, the heterogeneity among the included studies, particularly in terms of geographical location, population characteristics, and outcome measures, complicates the ability to draw solid conclusions. The lack of standardized protocols across studies may have introduced bias and limited the comparability of results. Second, the absence of randomized controlled trials (RCTs) in this review and the small sample size of almost all the studies included underscores the rarity of this condition and highlights the variability in treatment approaches across different referral centers as well as the influence of the surgical team’s expertise, potentially affecting the representativeness and generalizability of the results. Third, many of the studies had short follow-up times, which may limit the interpretation of the findings, increasing in turn the likelihood of bias. Particularly, this issue makes it difficult to assess long-term outcomes and recurrence rates. Finally, the scope of this review was restricted to three specific surgical approaches, which, even if most are performed, may not fully capture the latest advancements in the field. Future research should aim to address these limitations by standardizing methodologies, increasing sample sizes, and incorporating more diverse populations to enhance the robustness and applicability of the findings.

Soon in the future, robotic-assisted surgery could represent a valuable tool in the treatment of skull base chordomas, offering enhanced precision and control in this challenging anatomical region and thus reducing the risk of damage to critical neurovascular structures, facilitating more complete tumor resections, and potentially leading to better patient outcomes. As robotic-assisted techniques continue to advance, they are becoming an integral part of modern chordoma surgery, improving both safety and effectiveness in these complex cases, as highlighted by some studies [72,73,74,75]. Furthermore, the development of advanced imaging techniques, particularly regarding nuclear medicine, could improve the characterization of these lesions, improve surgical planning and potential adjuvant chemo- and radiotherapies, and monitor recurrences in follow-up [76].

## 5. Conclusions

It is still a subject of debate concerning how to select the most suitable surgical approach for chordomas—the approach that can offer an optimal EOR while preserving neurological functionality and quality of life and can ensure the lowest possibility of intra- and postoperative complications. According to the most updated literature findings, the most suitable surgical approach has to be adjusted to the specific size, location, and extension of the tumor on a case-by-case evaluation.

## Figures and Tables

**Table 1:** Demographic and surgical approaches of the studies included in the systematic review. Abbreviations: EEA—Endoscopic Endonasal Approach; mo—Months; RT—Radiotherapy; UK—United Kingdom; N/R—Not Reported; USA—United States of America; EEEA—Extended Endoscopic Endonasal Approach; ETISA—Endoscopic Transnasal Interseptal Approach; PBT—Proton Beam Therapy; IMRT—Intensity-Modulated Radiation Therapy.

Authors, Year	Country	N of Patients	Mean Age (Years)	Approach	Follow Up (mo)
Arbolay et al., 2009 [7]	Cuba	2	31	EEA	6
				
Baldassarre et al., 2021 [8]	Italy	8	35,75 (range 14–77)	5 EEA	24
				3 EEA + posterior approach	
Butenschoen et al., 2021 [9]	Germany	42	53 (range 29–69)	EEA	37 (range 6–60)
Ceylan et al., 2021 [10]	Turkey	72	41.67 ± 17.785	EEA + transmaxillar (3) EEA + transpterygoid (13) EEA + transcavernous (38) EEA + transodontoid (7)	31 ± 0.7 (range 6–143)
Chibbaro et al., 2013 [11]	France	54	49	EEA	34
Choi et al., 2010 [12]	UK and Germany	97	Range 41–60	40 standard TOAs 16 TOAs + soft palate split 44 “open door” maxillotomy 9 transmandibular 4 midface degloving procedures	50.4 (median 41; range 3–186)
Dehdashti et al., 2008 [13]	Canada	12	49.4	EEA	16
Fatemi et al., 2008 [14]	USA	14	47 ± 15	EEEA	20 (2 multiple operations and 2 tumor recurrences, 9 RT)
Frank et al., 2006 [15]	Italy	11	59.3	EEEA	27 (range 15–69)
Fraser et al., 2010 [16]	USA	7	52 ± 18	EEA	18
Garzaro et al., 2015 [17]	Italy	9	57.4	EEA	9.27 (range 3–19)
Holzmann et al., 2010 [18]	Germany	13	45.5	EEA	18 (range 2–48)
Kassam et al., 2008 [19]	USA	4	25.75 (range 16–36)	EEEA	N/R
Kim et al., 2016 [20]	South Korea	42	48.7 (range 10–72)	38 EEAs 3 EEAs + transpterygoid 1 transodontoid	N/R
Kong et al., 2021 [21]	South Korea	50	N/R	EEA	34.5 ± 8.2
Koutourousiou et al., 2012 [22]	USA	60	41 (range 4–84)	EEA	17.8 (29 patients received RT postop, 9 tumor progressions, 21 disease-free)
Kutlay et al., 2018 [23]	Turkey	106	N/R	EEA	28 (6–48)
Li et al., 2020 [24]	China	1886 (217)	N/R	EEEA	42,5 (chordoma recurred in 97 patients)
McDowell et al., 2021 [25]	USA	20	12.2 (range 4–18)	14 EEAs 6 EEAs + open transcervical	59 (range 1–166)
Menezes et al., 2014 [26]	USA	5	Range 5–14	3 TOA resections + fusions 1 initial C2-4 laminectomy and tumor resection, then vertebral artery embolization and C2-C4 lateral fusion, and extrapharyngeal excision w/corpectomy 1 lateral extrapharyngeal approach + tumor resection	8 ± 1.8 (range 2–16)
Messerer et al., 2016 [27]	Switzerland	3	46.3	EEEA	N/R (2 free of disease, 1 controlled residue)
Metcalfe et al., 2021 [28]	UK	11	53 (23–81)	EEA	7 years (9 patients PBT, 1 IMRT)
Nation et al., 2018 [29]	Malaysia	5	23.2 (range 11–57)	EEA	N/R
Passeri et al., 2023 [30]	France	210	47.6 ± 17.0	142 EEAs 15 TOAs 15 EEAs + open 7 MTS 31 open	59.2 ± 51.9 (range 3.0–369.1, median 43.4) postop RT in 163 patients
Ramm-Pettersen et al.,2017 [31]	Norway	6	61 (15–65)	EEA	91 (48–158)
Quon et al., 2019 [32]	USA	42	12.5 (range 4–18)	EEA	46 (range 1–120)
Rahme et al., 2018 [33]	USA	17	48.06	EEA	63,4 (tumor recurrence after RT in 5 patients)
Saito et al., 2012 [34]	Japan	6	59 (range 42–72)	EEA	15.83
Shin et al., 2015 [35]	Japan	32	55 (range 17–72)	ETISA	Range 3–6
Shkarubo et al., 2018 [36]	Russia	103	N/R	EEA	N/R
Schur et al., 2022 [37]	USA	78	N/R	38 EEA 40 open	66,56
Solares et al., 2005 [38]	USA	3	N/R	EEA	13
Soloperto et al., 2019 [39]	Italy	9	61	EEA	24.9 (range 7–36)
Spiessberger et al., 2022 [40]	USA	8	43,9	EEA	N/R (4 RT + 3 stereotactic radiosurgery)
Stippler et al., 2008 [41]	USA	20	44.35 (range 4–76)	EEA	13 (range 1–45) postop RT in 8 patients
Tan et al., 2012 [42]	Australia	14	48.5	EEA	41.5 (range 3–104)
Taniguchi et al., 2012 [43]	Japan	4	56,75	EEA	21.3 (all patients symptom-free)
Vellutini et al., 2014 [44]	Brazil	38(26)	46 (range 6–79)	EEA	Range 6 mo–11 years
Xin et al., 2022 [45]	China	3	N/R	EEA	7.59
Yano et al., 2014 [46]	Japan	6	N/R	EEA	23.1
Yang et al., 2011 [47]	China	2	49.5	Anterior retropharyngeal	32.5
Yoo et al., 2023 [48]	South Korea	17	38.7 (range 8–59)	11 EEAs 6 EEAs + open	66.7 (range 9–132)
Yousaf et al., 2019 [49]	UK	10	49	EEA	39.5
Zacharias et al., 2019 [50]	India	7	51	6 EEAs 1 combined EEA + TOA	24
Zhang et al., 2008 [51]	China	7	39.42	EEA	21.4 (range 3–39)
Zhong et al., 2021 [52]	China	102	48.75	Combined anterior retropharyngeal + posterior approach	N/R
Zoli et al., 2018 [53]	Italy	6	46,8	EEA	18 ± 7.3
Zoli et al., 2018 [54]	Italy	65	48 (9–80)	EEA	48
Zweckberger et al., 2020 [55]	Germany	50	39	EEA: 15 primary and 9 recurrences TOA: 3 primary and 2 recurrences	N/R (20 postop RT + 1 chemotherapy on primary surgery, 15 postop RT + 6 chemotherapy for recurrent surgery)

**Table 2:** Technical aspects, outcomes, and complications related to the surgical approach of the studies included in the systematic review. Abbreviations: CSF—Cerebro-Spinal Fluid; GTR—Gross Total Resection; N/R—Not Reported; STR—Subtotal Resection; NTR—Near—Total Resection; PR—Partial Resection; CN—Cranial Nerve; ICA—Internal Carotid Artery.

Authors, Year	Symptoms	Clivus Site	Cervical Level	Intradural Extension	EOR	Postop CSF Leak	Other Complications	Time Surgery	Hospitalization (Days)	Recurrency
Messerer et al., 2016 [27]	2 neck pain and dysphagia 1 CSF leakage and meningitis	3 upper	No	No	3 GTR	No	No	Primary surgery	N/R	N/R
Zweckberger et al., 2020 [55]	24 double vision 17 headaches 7 vertigo 9 visual acuity deteriorations 9 dysphagia 6 insecure gait 4 ptosis 1 coordination disorder	12 upper, 26 middle	N/R	17	12 GTR 28 STR	9	2 hemorrhages 3 strokes 2 hydrocephalus	29 primary surgeries 41 surgeries upon recurrence	N/R	N/R
Fatemi et al., 2008 [14]	8 diplopia (VI nerve palsy) 3 V nerve palsy 7 headaches 2 acuity visual losses 2 unsteady gait 2 spontaneous CSF leaks	9 upper, 5 middle	No	7	6 GTR 6 NTR 2 STR	8	1 transient diabetes insipidus	Primary surgery	N/R	N/R
Vellutini et al., 2014 [44]	16 VI nerve palsy 9 headaches	26 lower, 2 middle	No	12	13 GTR 7 STR 6 PR	6	2 meningitis 1 stroke	N/R	N/R	N/R
Rahme et al., 2018 [33]	12 diplopia 8 headaches 3 cranial nerve palsy 2 paresthesia 1 neck pain 1 unsteady gait 1 tinnitus 1 dysphagia 1 coma 1 decreased smell 1 airway obstruction	3 lower	1	9	9 GTR	6	5 cranial nerve palsy 3 meningitis 2 strokes	Primary surgery	N/R	N/R
Kassam et al., 2008 [19]	2 headaches 1 VI nerve palsy 1 III nerve palsy	N/R	N/R	N/R	3 GTR 1 STR	3	No	N/R	N/R	N/R
Koutouroulsiou et al., 2012 [22]	28 VI nerve palsy 17 headaches 4 nasal obstructions 5 III nerve palsy 5 trigeminal neuralgia 5 X nerve palsy 5 XII nerve palsy	23 lower, 21 middle, 7 upper	7	29	29 GTR + 11 GTR in previously treated patients 4 NTR + 5 NTR in previously treated patients 2 STR + 4 STR in previously treated patients 5 partial in previously treated patients	12	4 cranial nerve palsy 2 ICA injuries 2 meningitis	35 primary surgeries 25 recurrent surgeries	4,5	N/R
Kutlay et al., 2018 [23]	N/R	N/R	N/R	N/R	3 GTR 2 STR	No	2 VI nerve palsy (1 transient)	N/R	Range 3–36 (median 5)	N/R
Kong et al., 2021 [21]	N/R	22 upper, 38 middle	N/R	N/R	53 GTR 5 STR	5	2 meningitis 3 VI nerve palsy	Primary surgery	N/R	N/R
Nation et al., 2018 [29]	1 VI nerve palsy	2 upper	N/R	No	N/R	No	1 velopharyngeal insufficiency	Primary surgery	N/R	N/R
Yousaf et al., 2019 [49]	3 VI nerve palsy 3 headaches 2 transient diplopia 2 III nerve palsy	8 upper, 2 middle, 1 lower + occipitocervical junction	1	No	4 GTR 4 NTR 2 STR	2	1 meningitis 1 hypopituitarism 1 diabetes insipidus	Primary surgery	N/R	N/R
Zoli et al., 2018 [54]	2 VI nerve palsy 4 XII nerve palsy 1 dysphagia 1 cervical pain	6 middle	6	4	2 GTR 4 STR	No	1 pneumonia	Primary surgery	N/R	N/R
Kim et al., 2016 [20]	20 VI nerve palsy 7 headaches 2 acuity visual impairments	17 upper, 8 middle, 17 lower	N/R	19	28 GTR 14 STR	7	1 VI nerve palsy	34 primary surgeries 8 recurrences	N/R	N/R
Shkarubo et al., 2018 [36]	78 oculomotor disorders 35 V nerve palsy 28 dysphagia 13 acuity visual impairments 9 hemiparesis 34 headaches 11 coordination disorders	N/R	N/R	N/R	67 GTR 22 STR 10 partial	N/R	N/R	Primary surgery	N/R	N/R
Zacharias et al., 2019 [50]	4 VI nerve palsy Headache (not specified numbers)	6 upper	1	7	N/R	1	No	5 primary surgeries 2 recurrences	5	N/R
Metcalfe et al., 2021 [28]	N/R	N/R	N/R	N/R	N/R	N/R	1	1 radiation toxicity and carotid stenosis 1 postop infection	N/R	N/R
Butenschoen et al., 2023 [9]	14 VI nerve palsy 9 IX nerve palsy 19 headaches	5 upper, 4 middle, 26 lower	No	N/R	N/R	N/R	N/R	N/R	N/R	N/R
Li et al., 2020 [24]	N/R	No	N/R	N/R	54 GTR 91 STR 57 partial resections (79–90%) 15 partial resections (<70%)	N/R	N/R	N/R	N/R	N/R
Chibbaro et al., 2013 [11]	43 VI nerve palsy 22 headaches 11 ophthalmoplegia 11 acuity visual impairments	22 upper	3	19	35 GTR 9 NTR 10 partial resections	4	5 meningitis	32 primary surgeries 22 recurrences	N/R	N/R
Spiessberg et al., 2022 [40]	2 headaches 1 dysarthria 1 VI nerve palsy 1 XII nerve palsy 1 V nerve palsy	5 upper, 2 middle	1	2	4 GTR	1	1 VI nerve palsy 1 panhypopituitarism	Primary surgery	N/R	N/R
Arbolay et al., 2009 [7]	2 headaches 2 VI nerve palsy	N/R	N/R	N/R	1 GTR 1 STR	No	No	Primary surgery	N/R	N/R
Ngu et al., 2021 [56]	5 headaches 2 diplopia	1 upper, 1 middle, 2 lower	1	2	4 GTR 1 STR	No	1 syndrome of inappropriate antidiuretic hormone	4 primary surgeries 1 recurrency	16,6	N/R
Ceylan et al., 2021 [10]	37 headaches 29 acuity visual impairments 10 nausea 15 diplopia 34 hypopituitarism	19 upper, 9 middle, 25 lower	4	21	47 GTR 25 STR	7	2 VI nerve palsy 4 hydrocephalus	N/R	N/R	N/R
Dehdashti et al., 2008 [13]	5 headaches 2 unsteady gait 6 diplopia 1 VI nerve palsy 1 lower cranial nerve deficit	8 upper, 4 middle	No	7	6 GTR 6 STR	4	1 IX nerve palsy 1 motor hemisyndrome 1 hydrocephalus 1 tension pneumocephalus	9 primary surgeries 3 recurrences	Median 8	N/R
Shin et al., 2015 [35]	N/R	N/R	N/R	N/R	14 GTR	No	4 transient amnesia and III nerve palsy	11 primary surgeries 7 recurrences	N/R	N/R
Fraser et al., 2010 [16]	2 diplopia 3 VI nerve palsy 2 III nerve palsy	5 upper, 2 middle	No	3	5 GTR 2 NTR	No	1 pulmonary embolism	Primary surgery	N/R	N/R
Solares et al., 2005 [38]	1 nasal obstruction 2 acuity visual impairments	2 upper, 1 middle	No	N/R	N/R	N/R	No	1 primary surgery 2 recurrences	2	N/R
Soloperto et al., 2019 [39]	N/R	4 upper, 2 middle, 2 lower	No	N/R	3 GTR 5 STR	No	No	5 primary surgeries 4 recurrences	11	3
Stippler et al., 2008 [41]	5 headaches 2 III nerve palsy 6 VI nerve palsy 3 ophthalmoplegia	5 upper, 8 middle, 12 lower	3	9	9 GTR 4 NTR 7 STR	5	1 brainstem hemorrhage 2 transient neurological deficits 1 ICA rupture	12 primary surgeries 8 recurrences	N/R	N/R
Frank et al. [15]	9 VI nerve palsy 5 headaches 1 dysphagia	11 upper	No	N/R	5 GTR 5 STR 1 partial resection	No	N/R	7 primary surgeries 4 recurrences	5	1
Garzaro et al., 2015 [17]	4 headaches 6 diplopia	4 upper, 1 middle, 4 lower	No	N/R	6 GTR 1 NTR 2 partial resections	2	1 V I nerve palsy 1 hypokaliemia	7 primary surgeries 2 recurrences	10	2
Holzmann et al., 2010 [18]	2 VI nerve palsy 2 III nerve palsy 3 V nerve deficits	N/R	N/R	N/R	11 GTR 1 NTR 1 STR	1	N/R	N/R	N/R	N/R
Xin et al., 2022 [45]	N/R	N/R	N/R	N/R	3 GTR	N/R	1 VI nerve palsy	Primary surgery	N/R	N/R
Zhang et al., 2008 [51]	3 diplopia 3 headaches 2 nasal obstructions 3 VI nerve palsy	7 upper, 6 middle, 8 lower	2	4	6 GTR 1 STR	No	1 subarachnoid hemorrhage	5 primary surgeries 3 recurrences	N/R	N/R
Quon et al., 2019 [32]	2 VI nerve palsy 2 headaches 1 diplopia	N/R	N/R	N/R	2 GTR	N/R	N/R	N/R	N/R	N/R
Yoo et al., 2023 [48]	8 diplopia 2 headaches 1 dysphagia 2 trigeminal neuralgia	4 upper, 5 middle, 6 lower	2	6	6 GTR 9 NTR 2 STR	1	N/R	12 primary surgeries 5 recurrences	N/R	N/R
Baldassarre et al., 2021 [8]	5 neck pain 2 VI nerve palsy 1 dysphagia 1 rhinolalia	4 lower	4	1	6 GTR 2 partial resections	1	1 XII nerve palsy 1 hydrocephalus 1 pulmonary aspergillosis	7 primary surgeries 1 recurrency	N/R	N/R
Zoli et al., 2018 [54]	12 acuity visual impairments 25 V nerve neuralgia 1 dysphagia 9 diplopia 4 hemiparesis 2 VII and VIII deficits	45 upper, 12 middle	No	25	47 GTR 28 STR 5 partial resections	2	2 ICA injuries 1 hematoma 7 VII nerve palsy	37 primary surgeries 28 recurrences	N/R	N/R
Taniguchi et al., 2012 [43]	2 VI nerve palsy	1 middle	No	1	4 GTR	1	1 VI nerve palsy	1 primary surgery 3 recurrences	N/R	N/R
Tan et al., 2012 [42]	5 headaches 4 VI nerve palsy 2 diplopia	N/R	N/R	N/R	7 GTR 7 STR	3	1 hydeocephalus 1 aspiration pneumonia	7 primary surgeries 7 recurrences	14	N/R
Passeri et al., 2023 [30]	88 diplopia 84 VI nerve palsy 64 neck pain 65 headaches 53 XII nerve palsy	50 upper, 95 middle, 65 lower	95	115	92 GTR 72 NTR 33 STR 13 partial resections	32	47 worsening cranial nerve palsy	166 primary surgeries 44 recurrences	N/R	N/R
Schur et al., 2022 [37]	N/R	34 upper, 31 middle, 37 lower	16	30	N/R	N/R	N/R	N/R	N/R	N/R
Saito et al., 2012 [34]	VI nerve palsy (not specified number)	5 upper, 1 middle, 4 lower	No	4	3 GTR 1 STR 2 partial resections	No	1 meningitis 1 hydrocephalus	6 primary surgeries	N/R	N/R
Ramm-Pettersen et al., 2017 [31]	5 diplopia 3 headaches 1 facial hypoesthesia	3 upper, 3 lower	N/R	2	3 GTR 1 NTR 2 partial resections	1	No	6 primary surgeries	N/R	N/R
McDowell et al., 2021 [25]	7 diplopia 6 headaches 4 swallowing difficulties	14 upper, 1 middle, 3 lower	2	14	14 GTR 6 NTR	3	2 VI nerve palsy 1 Horner’s syndrome 1 epidural hematoma	15 primary surgeries 4 recurrences	N/R	N/R
Yano et al., 2014 [46]	6 VI nerve palsy	N/R	N/R	N/R	4 GTR 2 STR	1	N/R	N/R	N/R	N/R
Choi et al., 2010 [12]	86% neck pain 18.6% myelopathy	N/R	N/R	N/R	N/R	6.2%	25% tumor recurrence 4.1% chest infection 3.1% meningitis 3.1% velopharyngeal incompetence 2.1% new cranial nerve palsy 1% wound infection—pharyngeal 3.1% sepsis 3.1% dysphagia 2.1% fixation failure 1% vertebral artery stroke	N/R	N/R	N/R
Menezes et al., 2014 [26]	100% neck pain 2 w/quadriparesis 1 w/swallowing difficulty and XI nerve palsy	1 middle	2 C2 1 C1 1 C2-C3 1 C1-C2 to clivus	1	GTR	N/R	No	Primary surgery	N/R	N/R
Yang et al., 2011 [47]	Neck pain Tetraparesis	No	2 C2-C3	N/R	GTR	1	1 swallowing difficulty (liquid)	N/R	N/R	Yes (13–18 mo postop)
Zhong et al., 2021 [52]	N/R	No	102 C2	N/R	21 en-bloc resections 81 total piecemeal resections	8	9 dysphagia 8 pneumonia 7 dyspnea 5 surgical site infections 2 hematoma 2 pharyngeal dehiscence 1 neurological deficit 1 symptomatic venous thromboembolism 1 cerebral infarction	Primary surgery	21.12 ± 6.32	N/R
